# Carboplatin-Pemetrexed in Treatment of Patients with Recurrent/Metastatic Cancers of the Head and Neck; Superior Outcomes in Oropharyngeal Primaries

**DOI:** 10.3389/fonc.2014.00362

**Published:** 2015-01-26

**Authors:** Binu Malhotra, Emily Light Bellile, Nghia Pham Trung Nguyen, Vicki Kay Fung, Matthew Slack, Rebecca Bilich, Silvana Papagerakis, Francis Worden

**Affiliations:** ^1^Division of Hematology Oncology, Department of Medicine, University of Michigan, Ann Arbor, MI, USA; ^2^Department of Public Health, University of Michigan, Ann Arbor, MI, USA; ^3^Department of Otolaryngology, Kresge Hearing Research Institute (KHRI), University of Michigan, Ann Arbor, MI, USA; ^4^University of Michigan, Ann Arbor, MI, USA; ^5^Department of Otolaryngology, University of Michigan, Ann Arbor, MI, USA

**Keywords:** head and neck cancer, carboplatin, pemetrexed, recurrent, metastatic, oropharyngeal neoplasms

## Abstract

**Background:** Platinum-based therapy in combination with 5-fluorouracil with cetuximab has shown the best survival in pts with recurrent/metastatic squamous cell carcinoma of the head and neck (R/M SCCHN). The purpose of this study is to evaluate the efficacy and tolerability of carboplatin, pemetrexed and to assess differential outcomes in patients with oropharyngeal primary and HPV related disease.

**Patients and Methods:** The charts of consecutive patients with R/M SCCHN were reviewed. All patients receiving at least one cycle of the two-drug regimen (pemetrexed 500 mg/m^2^, carboplatin area under the curve of five intravenously), were included for assessment of response, safety, toxicity, and survival.

**Results:** A total of 86 patients received this regimen between January 2008 and December 2012, of which, 63 were included in this analysis. Forty-one percent (26) of the patients had cancers of the oropharynx, and of those, 50% had HPV-positive disease, 32% (20) had cancers of the larynx, and 24% (15) of the oral cavity. Median number of cycles administered was 4 (range 1–14 cycles) with 50% of the patients receiving four or more cycles. Half the patients achieved stable disease as their best response, 8% (5) attained a partial response, 24% progressed on therapy, and the remaining patients (12) could not have their response assessed. On the basis of Kaplan–Meier analysis, median progression-free survival (PFS) was 5.1 months (95% CI 3.2, 6.2) and median overall survival (OS) was 9.4 months (95% CI 4.3, 13.1). Among pts with oropharyngeal primary (*n* = 26), median PFS was 6.4 months (95% CI 2.8, 7.9) and median OS was 16.6 months (95% CI 9.6, 19.5). Among HPV+ pts (*n* = 13), median PFS was 7.0 months (95% CI 4.8, ne) and median OS was 17.1 months (95% CI 11.2, 21.7).

**Conclusion:** Combination carboplatin-pemetrexed is an effective and well-tolerated treatment, associated with a median PFS of 5.1 months and a clinical benefit in at least 57% of the patients treated.

## Introduction

To date, there are few therapies that provide significant survival benefit in patients with recurrent/unresectable or metastatic squamous cell cancer of the head and neck (R/M SCCHN). The current standard of care for patients with good performance status (PS) is platinum-based chemotherapy, typically in combination with 5-FU or taxol. Median survival reported with these combinations is in the realm of 7–8 months (1). More recently, the median survival was noted to be increased to 10 months with addition of cetuximab to a chemotherapy doublet backbone. However, this increased survival came at the cost of adding a third agent to the treatment regimen and the possibility of additional toxicity (2). Pemetrexed is an inhibitor of thymidylate synthase, as well as other folate-dependent enzymes now used in management of various malignancies (3, 4). Pemetrexed was initially approved in 2004 as a single agent for second line treatment of metastatic non-small cell lung cancer (NSCLC) (5). It has shown efficacy in treatment of patients with non-squamous NSCLC, now considered standard of care for upfront management of metastatic disease in combination with carboplatin (6, 7). It is very well tolerated even when used for a prolonged period of time as a maintenance therapy in patients with NSCLC (8). This drug has been evaluated in setting of head and neck cancers, the results, however, have not been consistent across studies (9–12).

In a recent phase III trial, Urba and colleagues compared pemetrexed in combination with cisplatin to cisplatin alone in patients with R/M SCCHN. While the combination of cisplatin plus pemetrexed did not result in improvement in the overall survival (OS) or progression-free survival (PFS) for all patients included in the analysis, the pre-defined subgroup analysis did demonstrate positive results among patients with a better performance status (PS) and patients with an oropharyngeal primary (12). In patients with PS 0–1, pemetrexed plus cisplatin significantly improved the OS (8.4 months compared to 6.7 months with cisplatin alone, HR 0.83, 95% CI 0.7–0.98, *p* = 0.026). This is likely due to the ability of this group of patients to better tolerate a combination regimen compared to patients with PS 2 or greater. Given the improved outcomes in patients with a better PS, many contemporary studies only include patients with a PS of 0–1.

We at our institution have been using pemetrexed in combination with carboplatin in patients with R/M SCCHN, who are candidates for salvage chemotherapy and have witnessed comparable outcomes with this regimen to standard of care; and improved tolerability. In this population with metastatic/recurrent disease, maintaining quality of life is of paramount importance. This regimen is associated with excellent tolerability and convenience, without a requirement for prolonged infusion (as needed with 5-Fluorouracil) or the risk of neuropathy as is associated with paclitaxel.

## Patient and Methods

### Eligibility

All patients treated for R/M SCCHN at the University of Michigan between Jan 2008 and Dec 2012 were identified and their medical records were reviewed. All patients who received at least one cycle of carboplatin-pemetrexed were included for assessment of response, safety, and tolerability. A total of 86 patients were treated with this regimen during this period. Eligibility for cytotoxic therapy and this regimen in particular was determined by the treating oncologist. Sixty-three of the 86 patients were included for this analysis. Patients with another concurrent malignancy and patients with a primary malignancy of the salivary glands, skin, sinus, or nasopharynx were excluded from the analysis.

### Study design and treatment

In this single-center retrospective review, the goal was to analyze the outcomes in all patients with SCCHN, treated with this novel regimen during the designated period, and to assess its efficacy and tolerability outside the clinical trial setting.

The primary endpoint of our analysis was to determine PFS and OS. Secondary endpoints included response rate, clinical benefit rate, effect of HPV status and primary site on survival, and assessment of adverse effects (grade ≥2). Relevant patient information was obtained from our Epic electronic medical record (EMR) system after appropriate approval by the Institutional Review Board. Records were reviewed starting from time of diagnosis to their last follow up or death. Baseline demographics were recorded, including other comorbidities. Treatment details, dose reductions, discontinuation, toxicities, hospitalizations, and date of last follow up, were all recorded.

Treatment included a two-drug regimen utilizing pemetrexed 500 mg/m^2^ and carboplatin area under the curve of five intravenously (every 3 weeks) until disease progression or in-tolerance to treatment. Folic acid supplementation (1 mg/day) was given during the entire course of treatment along with Vitamin B12 (1000 μg intramuscularly), which was started before the initiation of chemotherapy and given every 9 weeks thereafter. Response was graded by the treating oncologist using the RECIST criteria (13) and verified by source documentation of radiographic readings. Computed axial tomography (CT) or rarely MRI was obtained after the first two cycles and subsequently every two to three cycles of chemotherapy; hence, every 6–9 weeks. Adverse effects were retrospectively graded from chart records using the NCI criteria (CTCAE) version 4.

### Statistical analysis

Descriptive statistics, such as median and range, were used to describe the patient population and follow up. Survival estimates were calculated using the Kaplan–Meier method. PFS was calculated from the time of initiation of therapy to the time of progression, death, or last follow up. OS was calculated from the time of treatment initiation to the time of death or last follow up. Patients receiving treatment at the time of last follow up had that as the censoring date for survival analysis.

## Results

### Patient characteristics

Of 86 patients treated with a combination of carboplatin-pemetrexed in the head and neck cancer clinic at our institution between January 2008 and December 2012, 63 were included for this analysis. Twenty-three patients were excluded because their primary tumor originated in the salivary glands, in the sinus, nasopharynx, or skin and one patient was excluded because of a concurrent small cell cancer of the lung.

Median age for this group of patients was 59 years (range, 32–80 years). Five percent of patients had a new diagnosis of metastatic disease, 30% had recurrent disease, and 65% had recurrent and metastatic disease (Table 1). ECOG PS was 0 in 19% (12) of the patients, 1 in 70% (44) of the patients, and 2 in 11% (7) of the patients at the time of treatment initiation. Fifty-eight percent of the patients were current or former smokers. Forty-one percent of the patients had an oropharyngeal primary of which 50% of the cancers were HPV related. Carotid involvement was present in 11% (7) of the patients and two patients died from massive bleeding during treatment.

**Table 1:** **Patient characteristics and disease characteristics**.

Characteristic	Number of patients
Median age, years (Range)	59 (32–80)
**Smoking history**	
Current/former smoker	37
Never/remote smoker	26
**Stage of the disease**	
Recurrent	19
Metastatic	3
Recurrent/metastatic	41
**Primary site**	
Oropharynx	26
Oral cavity	15
Larynx	20
Unknown	2
**Sites of involvement**	
Carotid involvement	7
Regional lymph nodes	40
Lungs	29
Liver	6
Brain	3
Bones	7
**HPV status among oropharynx ca**	
HPV positive	13
HPV negative	4
HPV unknown	9

Of 63 patients, 77% had prior chemoradiation for management of locally advanced disease and 21% had been treated with at least one line of chemotherapy in the palliative setting, prior to the regimen in question (Table 2). Another 8% of the patients received chemotherapy adjuvantly as part of a clinical trial and 5% were treated with induction chemotherapy.

**Table 2:** **Treatment characteristics**.

Characteristic	Number of patients
**Prior radiation therapy**	
Yes	13
No	50
**Prior surgery**	
Yes	35*
No	28
**Prior chemoradiation**	
Yes	49
No	14
Prior chemotherapy** (palliative chemo)	
1 Line	10
2 Lines	3
**Number of cycles treated with**	
<4 cycles	30
>4 cycles	33
**Subsequent lines of treatment**	
1 Line	20
2 Lines	7

*Four of the 35 surgeries were in fact metastatectomies. One patient had prior surgery and a metastatectomy as well.

**Another five patients had received chemotherapy in the adjuvant setting as part of a clinical trial.

### Treatment characteristics

All patients had a minimum follow up for at least 12 months (range 12–48 months). None of the patients included in this analysis were on this regimen at the time of data collection. Median number of cycles administered was 4 (range, 1–14 cycles). Fifteen patients (24%) progressed on this treatment regimen and did not receive any more than two cycles.

Two patients passed away from a carotid bleed after just one cycle of treatment. One patient refused further therapy after one cycle for unclear reasons. One patient refused further therapy after three cycles because of fatigue. One patient transferred care after just one cycle of therapy. One patient had suicidal thoughts and treatment was with held and patient passed away shortly thereafter. Five of seven patients with PS of 2, demonstrated clinical deterioration with treatment and just received one to two cycles of treatment. Six patients received a predetermined number (a total of four cycles) of carboplatin-pemetrexed after a definitive therapy to the metastatic site of disease. Ten patients had therapy discontinued to provide a chemo holiday or break from therapy in light of stable disease or partial response.

### Response and survival analysis

Response was assessed by the treating oncologists based on radiological studies. Forty-nine percent (31) of the patients achieved stable disease as their best response, 8% (5) of the patients demonstrated a partial response, and 24% (15) of the patients progressed on this therapy. Response could not be assessed in the remaining 12 patients – 2 patients with carotid involvement died from a massive bleed after just one cycle of therapy. Six patients had some form of clinical deterioration, which was not directly attributed to the cancer or the treatment but was likely related to both and an underlying poor PS and their response to treatment was not assessed radiologically. One patient was lost to follow up and another one transferred care, after just one cycle of therapy. One patient refused further treatment after one cycle and another patient had treatment with held because of suicidal ideation and these patients did not have a response assessment. On the basis of Kaplan–Meier analysis, median PFS was 5.1 months (95% CI 3.2, 6.2) and a median OS was 9.4 months (95% CI 4.3, 13.1). Among patients with the oropharynx as the primary site of disease (*n* = 26), median PFS was 6.4 months (95% CI 2.8, 7.9) and median OS was 16.6 months (95% CI 9.6, 19.5).

In patients with cancers of the oropharynx that were positive for HPV, median PFS was 7.0 months (95% CI 4.8, ne) and median OS was 17.1 months (95% CI 11.2, 21.7). Forty-three percent of the patients moved on to receive another line of therapy upon disease progression.

### Survival after metastatectomy

Four patients underwent surgical resection to the oligo-metastatic disease, i.e., metastatectomy. Three out of four patients that underwent surgical metastatectomy for lung metastasis were alive at the time of follow up for 2 years from the time of therapy. All three patients had an oropharyngeal primary.

Another patient who had primary tumor of the oral cavity and underwent metastatectomy was noted to be deceased 12 months later. Another patient had definitive radiation to the mediastinal lymph node, which was the only site of metastatic disease; however, he was lost to follow up and survival can not be estimated.

### Toxicity

Grade 3 and 4 toxicities attributable to the regimen included fatigue (14%), neutropenic fever (5%), and hospitalization for infections/dehydration in another 16% of the patients. Hospitalization for various causes was mostly limited to patients that started out with a poor PS.

## Discussion

In our series, the combination of carboplatin and pemetrexed was noted to be active and tolerable in patients with metastatic or recurrent cancers of the head and neck. Even though this treatment was not associated with dramatic responses, it did stabilize the disease in about 50% of the patients. The PFS and OS seen with this regimen are encouraging and in the realm of what may be expected with standard of care. (2). In addition, this regimen is very well tolerated, fatigue being the only common adverse effect, especially in patients starting out with a normal PS. The tolerability of this regimen has been very well demonstrated in patients with NSCLC as well (8). In our experience, outcomes were particularly good in patients with cancers of the oropharynx and in those with HPV positivity, which is also in concordance with the published literature (10, 14).

The relatively modest response rates may be due to the refractory nature of disease in patients treated with this regimen. As already detailed above, 77% of the patients had prior treatment with chemoradiation and 34% of the patients had been treated with some type of chemotherapy prior to this treatment. As is noted in a study by Argiris et al., cancers of the oral cavity and hypopharynx, prior radiation treatment, and an ECOG PS of 1 (versus 0) are independent unfavorable predictors of objective response (14). Urba et al. had published the results of a well-conducted phase III study comparing cisplatin versus cisplatin + pemetrexed. The trial was overall a negative study; however, a pre-defined subgroup of patients with oropharyngeal primary and a good PS did confirm the superiority of this combination over cisplatin alone. Despite that, this regimen is unlikely to be ever investigated again in a systematic fashion. At our institution, we have extensive experience with this regimen given the excellent tolerability and comparable outcomes to the standard of care. Most of our experience is with carboplatin (not cisplatin) in combination with pemetrexed, which may account for better tolerability and our ability to treat until disease progression, which may in fact make this combination more effective.

We at our institution also treat oligo-metastatic disease aggressively, which was observed in this retrospective review as well. Four patients underwent a surgical metastatectomy and three of those four patients that had an oropharyngeal primary, were alive at their last follow up, 24 months from the time of treatment. This phenomenon has recently been reported by other institutions as well (15).The long-term survival of patients with oligo-metastatic disease may have impacted the OS for the entire group of patients with oropharyngeal disease. Also, 27 patients received another line of therapy, of which seven received two subsequent lines of therapy at time of disease progression. Of these, 14 patients had an oropharyngeal primary, once again accounting for the improvement in OS.

## Conclusion

Carboplatin-pemetrexed is a feasible, well-tolerated, and effective regimen in treatment of cancers of the head and neck. There is also a logistic advantage over other treatment regimens, convenience being a major factor for many of our patients.

Since the phase III study by Urba et al. was a negative study, another large study in this population is unlikely, and we strive to publish our experience and present this as another option in management of recurrent metastatic disease.

## Conflict of Interest Statement

The authors declare that the research was conducted in the absence of any commercial or financial relationships that could be construed as a potential conflict of interest.
